# 

**Published:** 1908-09-05

**Authors:** 


					71 rmir
? ;uR?Cw COiiitai.Of
SEP !4. :'jCb
r/\j
I |h closed!?
Telegraphic Address I THE MODERN S. ?al^hone Number :
Ospedaio, London. MEDICAL JOURNAL. ir?"Gerr?d.
New series. No. 78, Vol. III. Saturday
No. 1150. Vol. XLIV. JAlUtfUAi,
Sept. 5tit, 1908.
PRICE THREEPENCE

				

